# RNA-seq sheds light on “who is doing what” in the coral *Porites lutea*

**DOI:** 10.1186/s40168-026-02414-9

**Published:** 2026-05-02

**Authors:** Kshitij Tandon, Juntong Hu, Francesco Ricci, Linda Louise Blackall, Mónica Medina, Michael Kühl, Heroen Verbruggen

**Affiliations:** 1 School of BioSciences, The University of Melbourne, Melbourne, VIC 3010 Australia; 2 Melbourne Integrative Genomics, The University of Melbourne, Melbourne, VIC 3010 Australia; 3 Department of Microbiology and Immunology at the Peter Doherty Institute of Infection and Immunity, The University of Melbourne, Melbourne, VIC 3010 Australia; 4 Department of Microbiology, Biomedicine Discovery Institute, Monash University, Clayton, VIC 3800 Australia; 5 Securing Antarctica’s Environmental Future, Monash University, Clayton, VIC 3800 Australia; 6 Department of Biology, University of California Los Angeles, Los Angeles, CA USA; 7 Marine Biological Section, Department of Biology, University of Copenhagen, 3000 Helsingør, Denmark; 8 Spanish Institute of Oceanography, Spanish National Research Council (CSIC-IEO), Joint Unit UPV-IEO, Gandia, Spain

## Abstract

**Background:**

The coral holobiont functions as a complex biogeochemical system, sustained by intricate metabolic exchanges between the host and its associated microbiome. While the taxonomic diversity of these communities is well documented, the specific metabolic roles and biogeochemical contributions of microorganisms across distinct coral compartments, particularly within the endolithic habitats, remain poorly understood. Using RNA-seq, we investigated the active microbiome of healthy stony coral *Porites lutea*, focusing on the coral tissue, the green endolithic algal layer (Ostreobium layer), and the deeper coral skeleton.

**Results:**

We identified distinct, metabolically active communities within these compartments and highlight substantial metabolic redundancy across carbon, nitrogen, and sulfur pathways. Our study provides the first transcriptomic evidence of *Ostreobium’s* ability to transfer fixed carbon to other holobiont members and the coral host. We highlight the critical roles of diverse coral holobiont members in nutrient cycling and maintaining homeostasis through scavenging of reactive oxygen and nitrogen species.

**Conclusions:**

This study provides a novel molecular-level understanding of the functional roles played by diverse coral holobiont members in their respective compartments and underscores that corals harbor distinct microbiomes with wide-ranging functions.

Video Abstract

**Supplementary Information:**

The online version contains supplementary material available at 10.1186/s40168-026-02414-9.

## Introduction

Coral reefs owe their success to the symbiosis between reef-building, stony corals (Scleractinia) [1–3] and a large diversity of closely associated bacteria, archaea, viruses, and microeukaryotes, collectively termed the coral holobiont [3–6]. These symbionts sensu lato encompass organisms spanning the full spectrum of host-microbe interactions, from mutualistic partners that enhance coral fitness to opportunistic pathogens, as well as commensals and other microorganisms whose ecological roles may shift along this continuum depending on environmental conditions. While the most well-characterized symbiosis in corals involves photosynthetic, endosymbiotic microalgae in the family Symbiodiniaceae residing in the gastrodermal tissues of the animal host [7, 8], a compendium of prokaryotes associated with corals now includes 39 bacterial and 2 archaeal phyla [9]. This diverse microbial assemblage is not only distinct between different compartments within the coral [10], but fine-scale mapping of the coral skeleton has revealed a stratified community across its depth [11–13]. In recent years, other microeukaryotes besides Symbiodiniaceae, including endolithic algae of the genus *Ostreobium*, fungi, and apicomplexans have also garnered attention [14, 15].

The diversity and composition of coral holobiont members have been investigated through metabarcoding of ribosomal marker genes (16S rRNA, 18S rRNA, ITS2) [7, 16–19] providing invaluable insights into the diverse and structured symbiont composition sensu lato within coral compartments [11, 12, 20, 21]. Combined culture-based and molecular approaches have expanded our understanding of the functions of coral holobiont members in maintaining coral health and resilience [20–24]. Symbiodiniaceae, in particular, play a crucial role by supplying corals with photosynthates, meeting a significant portion of their energy demand [25]. Coral-associated bacteria and archaea have been implicated in a myriad of functions essential for sustaining coral health and resilience, including stress alleviation through scavenging of reactive oxygen and nitrogen species (ROS and RNS), protection against pathogens and nutrient cycling [20–22, 24, 26–28]. Coral skeleton-dwelling endolithic green algae of the genus *Ostreobium,* which form conspicuous green bands beneath the coral tissue, have also been suggested to support corals by providing photosynthates during coral bleaching [29–31], but we lack a detailed understanding of the metabolic interaction between the coral animal and *Ostreobium* [14].

Most of our present knowledge on the role of microbiota for coral function and health comes from amplicon and metagenomic sequencing studies, with potential functions inferred using correlative, bioinformatic analyses. Despite these advances and the extensive information now available on the gene-encoded functional potential of microbiome and microeukaryotic members through genomic and metagenomic studies, a significant knowledge gap persists regarding actual microbial metabolic activity and its spatial organization within the coral holobiont. Genomic data alone cannot predict the actual contributions of specific functions by symbionts in situ, as genes encoding these functions may be variably expressed or not expressed at all. RNA-seq enables the analysis of the pool of actively expressed genes in an environmental sample, highlighting the ecophysiologically relevant processes occurring at the time of sampling [32]. Therefore, taking a transcriptomics view can provide insights into which community members perform which functional roles.

Obtaining a molecular-level understanding of the functional roles of holobiont members is crucial for characterizing the fundamental biological processes that sustain these complex ecosystems. While recent research has highlighted the potential for microbiome manipulation to mitigate stress, we lack a comprehensive baseline of how metabolic processes are divided across the diverse eukaryotic and prokaryotic members of the coral holobiont [2, 33, 34]. Addressing this gap is essential for moving beyond taxonomic descriptions toward a predictive understanding of coral biogeochemistry. By identifying active metabolic pathways and their spatial organization, we can better define physiological boundaries within the coral holobiont and the mechanisms by which its internal nutrient economics are maintained [2, 13, 33].

In this study, we sought to answer the fundamental question of ‘who is doing what’ in a healthy coral holobiont across different compartments and map the functional architecture of its members. Using RNA-seq, we identified the active symbiont community of the coral *Porites lutea*, characterizing their specific activities and locations within the coral, from the tissue to the endolithic algal layer (*Ostreobium* layer) and deeper coral skeleton. We focused on key nutrient cycling pathways, including carbon fixation (C), photosynthesis (P), nitrogen (N), and sulfur (S) metabolism, sugar export, and the scavenging of reactive oxygen and nitrogen species (ROS and RNS).

## Methods

### Coral sampling and colony fragmentation

**Fig. 1:**
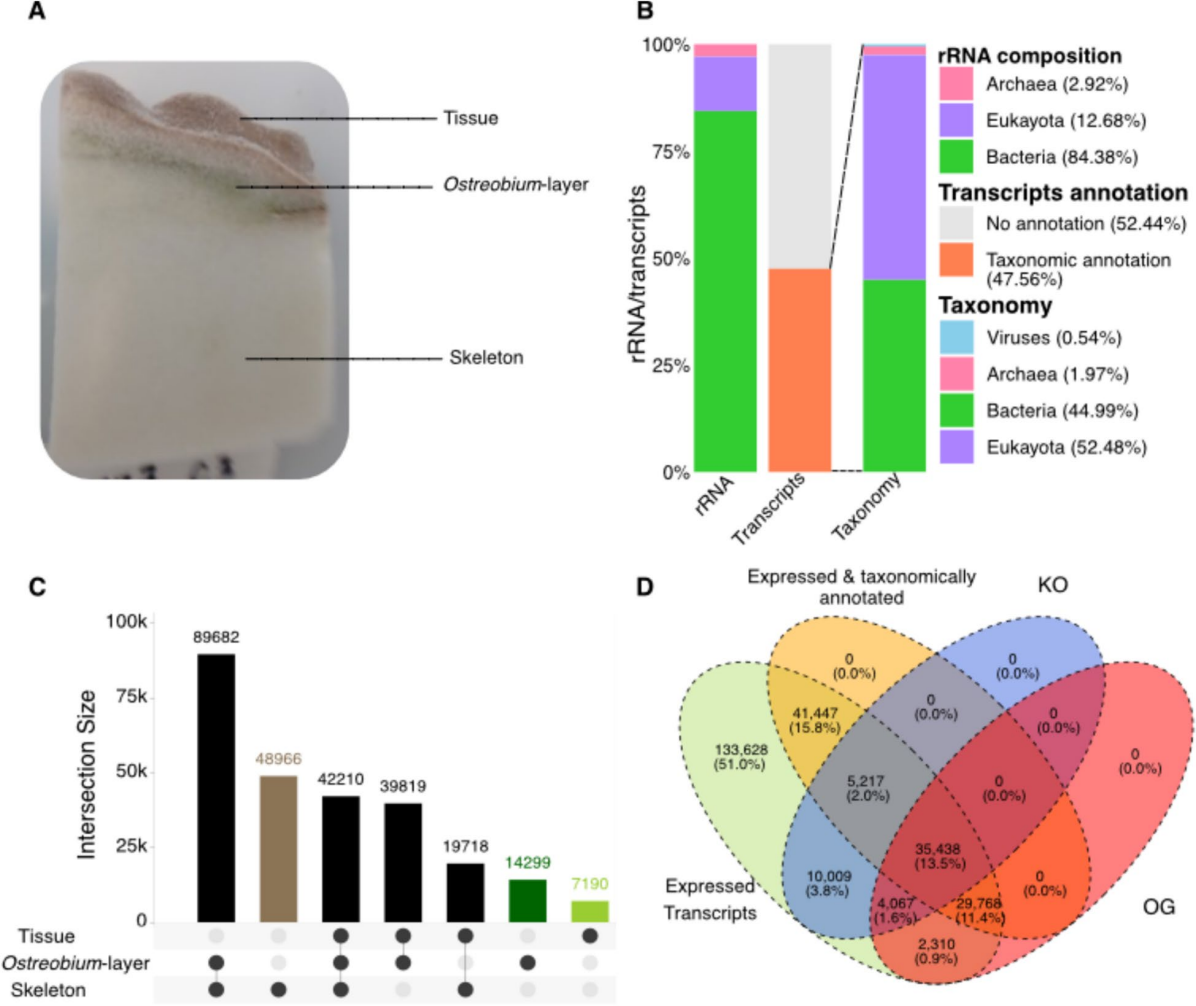
Taxonomic and functional overview based on rRNA and transcriptome data. **A** illustration of a coral fragment depicting the three compartments: tissue, *Ostreobium *layer and skeleton. **B** Bar plots showing overall community composition based on rRNA and transcriptome data. **C** UpSet plot illustrating shared and unique transcripts across different coral compartments. Colored bars indicate transcripts unique to a single compartment (tissue: light green; *Ostreobium *layer: dark green; skeleton: brown) while black bars represent transcripts shared between two or more compartments and **D** Venn diagram showing counts and percentage of transcripts which are deemed expressed, expressed and taxonomically annotated, annotated at Kegg Orthologs (KO) and at Orthologous groups (OG) levels

Fragments from six healthy-looking, *P. lutea* colonies, spaced at least 15 m apart, were collected at low tide (1–2 m depth) from the research zone of the Heron Island reef flat, southern Great Barrier Reef (23°44′S, 151°91′E), in October 2022. The health status of each colony was assessed in situ based on uniform pigmentation, full tissue coverage, and a complete absence of visible lesions, macroscopic competition with filamentous algae, or recent mortality within the sample area. Four fragments (from colonies we numbered 1, 3, 4, and 5) were collected during daytime, while two (colonies 6 and 7) were collected 5–6 h after sunset. All collected colonies exhibited a visually discernible endolithic green band (*Ostreobium* layer) in the coral skeleton 10–50 mm below the coral tissue layer (Fig. 1A). The fragments were collected using a sterile hammer and chisel and were placed in separate sterile zip-loc polyethylene bags in seawater and flash frozen in liquid nitrogen immediately upon collection. A 5-mm-thick slice was cut from the frozen samples, perpendicular to the vertical growth axis, using a diamond saw with a continuous flow of sterile filtered (0.22 µm) seawater. This seawater served primarily as a lubricant and coolant to dissipate frictional heat and prevent the thawing of the calcium carbonate matrix. Sectioning was performed rapidly (under 60 s per slice) using large skeletal fragments with high thermal mass, ensuring that the internal core of the material remained frozen throughout the procedure. Slices were immediately re-submerged in liquid nitrogen post-sectioning to maintain the flash-frozen state. Subsequently, sterilized pliers were used to isolate material from three distinct compartments: the coral tissue, the *Ostreobium* layer, and the deeper skeleton below the green band. To avoid cross-contamination at the interfaces, fragments were sampled specifically from the center of each layer. Throughout the fragmentation process, the samples were kept cryogenically stable by repeated immersion in liquid nitrogen. Each layer was stored at − 80 ºC, transported to the University of Melbourne on dry ice, and processed immediately upon arrival.

### Total RNA extraction

For RNA extraction, samples of individual layers were ground to a fine powder in liquid nitrogen using pre-cooled mortars and pestles. Approximately 200 mg of powdered tissue samples and between 300 and 500 mg of powdered *Ostreobium *layer and skeleton layer were used for RNA extraction with 1 mL of pre-cooled TRIzol reagent (Invitrogen, USA). Total RNA was extracted following the manufacturer’s protocol with some modifications (see Supplementary data file 1 for details). Quantification of total RNA was conducted using the Qubit RNA HS Assay kit (Thermo Fisher Scientific, USA). Total RNA yields were generally consistent across the tissue and *Ostreobium *layers; however, skeletal samples consistently exhibited lower concentrations (typically < 20 ng/µL). These lower initial yields are characteristic of the low-biomass skeletal matrix and remained within the functional range for high-sensitivity Illumina library preparation. All samples were retained for processing downstream.

### RNA sequencing and data-preprocessing

We did not perform any ribosomal RNA depletion due to the dual aim of sequencing mRNA and rRNA from both eukaryotes and prokaryotes simultaneously and profiling the transcriptionally active microbial and micro-eukaryotic community composition. Total RNA libraries were prepared for 36 samples (6 colonies × 3 layers × 2 replicates per layer) and sequenced on the Illumina NovaSeq platform by Azenta (Suzhou, China). Sequencing yielded approximately 23 million reads per sample with 2 × 150 bp reads (Supplementary Table S1).

Raw reads were quality checked and trimmed using FastQC v0.11.9 (https://www.bioinformatics.babraham.ac.uk/projects/fastqc) and Trimmomatic v0.39 [34], respectively. Trimmomatic parameters were as follows: *TruSeq3-PE.fa:2:30:5 HEADCROP:5 SLIDINGWINDOW:4:20 MINLEN:50*. Quality-filtered reads were first used to identify and filter out rRNA reads using SortMeRNA v4.3.6 [35] with Silva v138.1 LSU and SSU (SILVA v138.1 LSU and SSU NR99 database, last accessed Feb 10, 2023) [36]. Reads identified as rRNA (Supplementary Table S1) were taxonomically classified using PhyloFlash v3.4 [37] (with *almost-everything*), which rapidly reconstructs the SSU rRNAs (NTUs, nearest taxonomic units) and taxonomically annotates them using Silva v138.1 NR99 SSU database. Reads identified as rRNA were removed from the raw-read set. Following rRNA removal, non-rRNA reads were aligned to the *P. lutea* reference genome (accessed from www.reefgenomics.org) using Bowtie2 v2.3.4 [38]. To ensure a high-purity downstream dataset, we used Samtools v1.17 [39] to strictly retain only read pairs, where both mates were unmapped to the host (*flag -f 12*). This filtering excluded all host-related and discordant pairs for downstream assembly.

High-quality, non-Rrna, and non-host paired-end reads (Supplementary Table S1) were co-assembled using Trinity v2.15.1 [40] with parameters *–min_contig_length 250 and –no_salmon*. Taxonomic annotation of assembled transcripts was obtained using the lowest common ancestor approach by performing *blastx* [41] searches against the NR database (last accessed April 2023) with diamond v2.1.6.160 [42], using an *e*-value cutoff of 1e−5 and processed through MEGAN6 community edition v6.24.20, using the *megan-map-Feb2022-ue.db* and meganising the *daa* file using *daa2info* [43]. This process was used to identify and remove any transcripts annotated as Scleractinia and other Eumetazoa. The remaining transcripts were quantified using RSEM (RNA-Seq by Expectation and Maximization) [44], implemented in Trinity through the utility script *align_and_estimate_abundance*.*pl*. This approach generated both gene and isoform expression profiles. Isoform-level expression data was used for the analysis. Expression values per transcript were averaged across replicates for each layer (Tissue, *Ostreobium* layer, and Skeleton).

### Community composition analysis

Taxonomic classification of NTUs assembled from rRNA reads was conducted to profile the abundance of transcriptionally active community members. NTUs classified as “Chloroplast” or “Mitochondria” were removed. Abundance values were converted to relative abundance for compositional analysis using barplots and principal coordinate analysis (PCoA) based on Bray–Curtis dissimilarity. We used the quantified and taxonomically annotated transcripts assembled from mRNA reads to profile the community composition encoding for different functional guilds based on the transcriptome data and compared them to rRNA-based analysis using the same approach. These analyses were performed using R packages phyloseq v1.42.0 [45], metagMisc v0.5.0 (https://github.com/vmikk/metagMisc) and ggplot2 v34.3 [46].

### Functional analysis

To ascertain functional roles, raw transcript count matrices were first normalized using the Trimmed Mean of the M (TMM) method to account for differences in library size and composition across samples [47]. The robustness of TMM normalization to extreme count values minimizes the influence of low-abundance transcripts on scaling factor estimation. Following normalization, transcripts with TMM values of greater than 0.1 and detected in at least two samples were retained as truly expressed. This post-normalization filtering step was used to remove i) transcripts likely arising from residual DNA or extremely low expression and ii) transcripts resulting from spurious read mapping. Transcripts deemed truly expressed were then grouped in a compartment-specific manner to obtain tissue, *Ostreobium *layer and skeleton-layer specific transcriptomes for downstream analyses. To predict coding regions, we processed compartment-specific transcripts through Trinotate v4.0.0 [48] and TransDecoder v4.0.0 (https://github.com/TransDecoder/TransDecoder). Candidate open reading frames (ORFs) were identified for each transcript, and we retained only the longest ORF (≥ 50 amino acids) for downstream protein prediction and functional annotation. This conservative approach was selected to maximize the reliability of functional profiling in a de novo context where assembly fragments often contain spurious or truncated ORFs. While bacterial transcripts can be polycistronic, prokaryotic operons are typically organized into functionally syntenic blocks. Consequently, the longest ORF on a transcript serves as a robust metabolic marker for that fragment; even in cases where adjacent genes in an operon are not captured, the metabolic signal of the transcript remains functionally representative of the pathway. This prioritization ensures a high-confidence, non-redundant protein set for functional profiling. The same criteria were applied to both eukaryotic and prokaryotic transcripts to ensure methodological consistency. Predicted proteins were functionally annotated using a combination of EggNOG mapper v2.1.1 [49] with the database eggnogDB v5.0.2 [50] with *e-value* cutoff 1e−5, ghostkoala [51] with database *c_family_euk* + *genus_prok* + *viruses* parameters and Interproscan v5.63–95.0 [52] with *e-value* cutoff 1e−5 to obtain a comprehensive understanding of functional orthologous groups (OGs), KEGG pathways, including Kegg Orthologs (KOs) and protein families (pfam). Metabolic markers for carbon-fixation (C), photosynthesis (P), nitrogen (N), and sulfur (S) metabolism were identified from a previous study [53]. The use of such targeted metabolic marker gene sets is a well-established approach for reconstructing metabolic potential and biogeochemical cycling in complex environmental microbiomes [54, 55]. Additionally, we focused on genes involved in sugar export and scavenging of ROS and RNS. Expression profiles of transcripts functionally annotated with marker KOs or pfams were plotted using heatmaps using ComplexHeatmap [56] in R.

## Results and discussion

### Corals harbor compartment-specific active symbiont community

One of our primary objectives was to compare the transcriptionally active microbial community (inferred from mRNA) with the total community (inferred from rRNA) across three distinct holobiont compartments: the coral tissue, the endolithic *Ostreobium *layer*,* and the underlying skeleton (Fig. 1B). As we processed two sub-replicates per layer, we first assessed the similarity between them using PCoA at both rRNA and transcript levels (Supplementary Fig. S1). PCoAs demonstrated high reproducibility, with sub-replicates from the same colony and compartment in proximity (Supplementary Fig. S1). Despite variations in total RNA yield among some samples (e.g., JHS3A and JH5SB), PCoAs demonstrated high reproducibility, with sub-replicates from the same colony and compartment clustering tightly (Supplementary Fig. S1). This indicates that the biological signature remained consistent across replicates, regardless of the absolute mRNA read recovery (Supplementary Table S1). The analysis revealed clear niche differentiation, with samples grouping primarily by compartment (Tissue, *Ostreobium *layer*,* and Skeleton) rather than by colony or time of collection. Given this high technical consistency and the dominance of the spatial signal over other variables, sub-replicate abundance values were averaged for all downstream analyses. For total community composition, we taxonomically analyzed 28,717 rRNA NTUs (6010 from tissue layer, 15,942 from *Ostreobium *layer*,* 26,712 from skeleton layer) belonging to Archaea (2.92%,–840 sequences), Eukaryota (12.68%,–3644 sequences), and Bacteria (84.38%,–24,233 sequences) (Fig. 1B). This observation offers essential insights into the diversity and composition of the coral holobiont. Unlike traditional amplification-based metabarcoding analyses, which are often biased [57], using rRNAs assembled from total RNA or metagenome sequencing based rRNA surveys delivers a more precise and comprehensive overview of the community composition [58–60]. Eukaryotes (Amorphea, SAR sub-categories, Archaeplastida) were the dominant groups in the coral tissue (Supplementary Fig. S2A) and the *Ostreobium *layer (Supplementary Fig. S2B), while the deeper skeleton was dominated by Bacteria (Proteobacteria, Planctomyceota, Desulfobacteria, and Cyanobacteria) (Supplementary Fig. S2C). Our study shows that bacterial diversity is highest in the skeleton, where the majority of bacterial NTU sequences were assembled, confirming previous reports of greater bacterial richness in the skeleton compared to tissue compartments [12, 17].

After initially evaluating NTUs based on rRNAs, we extended our analysis to the remaining RNA sequence annotations. To profile active microbial community composition, we used a curated set of 371,193 taxonomically annotated transcripts, selected from a total of 780,431 assembled sequences. After excluding 111,367 transcripts belonging to Scleractinia, Chordata, and Eumetazoa, 259,826 (69.99%) transcripts remained. These transcripts represent Eukaryotes (52.48%,–136,366), Bacteria (44.99%,–116,900), Archaea (1.97%,–5131), and viruses (0.54%,–1429) (Fig. 1B) and revealed a high relative abundance of Symbiodiniaceae in coral tissue (Supplementary Fig. S3A). Green algae, Streptophyta, and Chlorophyta, dominated the *Ostreobium *layer and deeper skeleton (Supplementary Fig. S3B and C). The high relative abundance of dinoflagellate (Suessiaceae: Symbiodiniaceae) expressed transcripts in coral tissue is unsurprising, as they represent the most transcriptionally active microeukaryotes in this niche (Supplementary Fig. S3A), providing the bulk of the coral's energy demands [61]. By delineating the SAR mRNA signal, we confirmed that while Symbiodiniaceae dominate the functional output in tissue, diverse Apicomplexa and non-symbiotic dinoflagellate transcripts remain present across all compartments, including the skeleton (Supplementary Fig. S3). The presence of dinoflagellates (specifically Symbiodiniaceae) within the skeletal matrix is supported by evidence that the skeleton serves as a refuge, harboring viable symbionts that can repopulate host tissue following bleaching events [14, 30, 62]. This skeletal association is further corroborated by recent work showing that the coral skeleton can serve as a long-term archive of symbiont communities [63]. Similarly, the detection of active Apicomplexan transcripts across all niches aligns with their recognition as ubiquitous members of the healthy coral holobiont, though their specific ecological roles remain to be fully characterized [15, 64]. Bacteria and fungi were present in all compartments, with bacteria as dominant members of the skeletal microbiome (Supplementary Fig. S3C). Proteobacteria, Cyanobacteria, and Actinobacteria were the dominant bacterial phyla in all coral compartments. Members of the phylum Proteobacteria are some of the most widespread coral microbiome members [9, 40, 65]. Cyanobacteria have also previously been identified as the dominant prokaryote group in the *Ostreobium *layer and deeper skeleton of *P. lutea* from the Red Sea [66]. Obtaining annotation for less than 50% of the total assembled transcripts highlights the immense diversity of uncharacterized symbionts associated with *P. lutea,* despite it being one of the best investigated coral species with several metagenomic and physiological studies [20, 21, 67, 68]. These findings align with earlier studies using the meta-transcriptomics approach to profile transcriptionally active microbiomes in other ecologically diverse systems, including soil [69–71], sponge [72], and ocean microbiomes [53, 73].

### Functional annotation of transcripts remains a bottleneck

Analysis of active metabolic pathways in a healthy coral holobiont revealed 261,884 truly expressed transcripts through TMM based normalization and filtering. These transcripts were distributed across the three investigated compartments, with the highest overlap observed between the *Ostreobium*-layer and deeper skeleton, which shared 89,682 (34.24%) transcripts. In contrast, the overlap between all three compartments comprised 42,210 (16.12%) transcripts. The deeper skeleton exhibited the highest level of niche-specific functional complexity, harboring 48,966 (18.70%) unique transcripts. Conversely, the coral tissue exhibited the lowest count of uniquely expressed transcripts, with only 7190 (2.75%) transcripts specific to that niche (Fig. 1C). To further assess functional potential, we performed comprehensive annotation of the total transcript pool (Fig. 1D). Within this pool, 133,628 (51.0%) transcripts were expressed but lacked both taxonomic and functional annotation. This “dark matter” spanned the entire holobiont, representing a chemically and spatially complex landscape shared by host-microbe interactions, biomineralization, and endolithic microbial activity, processes that remain poorly understood across different niches in the coral holobiont [3, 74]. As genomic resources of coral-associated microbes and host expand, these currently unannotated transcripts from the tissue, *Ostreobium*-layer, and skeleton will likely yield novel metabolic pathways and niche specific markers. Characterizing this vast, unmapped protein space is essential for moving beyond established models and capturing the full metabolic and structural complexity of the coral holobiont [74–76]. The relatively low functional annotation observed in our dataset, where only 41,447 (15.8%) transcripts were successfully taxonomically and functionally annotated, is consistent with recent studies on global oceans, which found less than 25% of assembled transcripts assigned to KOs [53]. However, we successfully mapped 35,438 (13.5%) transcripts to all three categories: taxonomic annotation, KO and OGs (Fig. 1D). Annotations based on COG revealed that signal transduction (Tissue 1782; *Ostreobium*-layer: 4602; Skeleton 6074), intracellular trafficking, secretion, and vesicular transport (Tissue 991; *Ostreobium*-layer 2623; Skeleton 3579), post-translational modification, protein turnover, and chaperones (Tissue 1900; *Ostreobium*-layer 5016; Skeleton: 6482) were among the five most abundant functional categories across all three compartments (Supplementary Fig. S4). While most transcripts remained unannotated, the prominence of these signaling and regulatory pathways indicates a transcriptionally active microbiome engaged in consistent cellular maintenance and environmental sensing. Although such broad functional groups represent essential 'housekeeping' processes rather than direct evidence of metabolic syntropy, their activity confirms that the different coral compartments support a specialized and physiologically active microbial community. To determine whether these microbes provide specific benefits to the host, further targeted research, such as stable isotope probing (SIP) to track carbon/nitrogen exchange or metabolic modelling to identify cross-feeding dependencies, is required to move beyond descriptive functional profiling toward a mechanistic understanding of coral holobiont interactions.

### Distinct photosymbionts drive carbon fixation in coral compartments

**Fig. 2:**
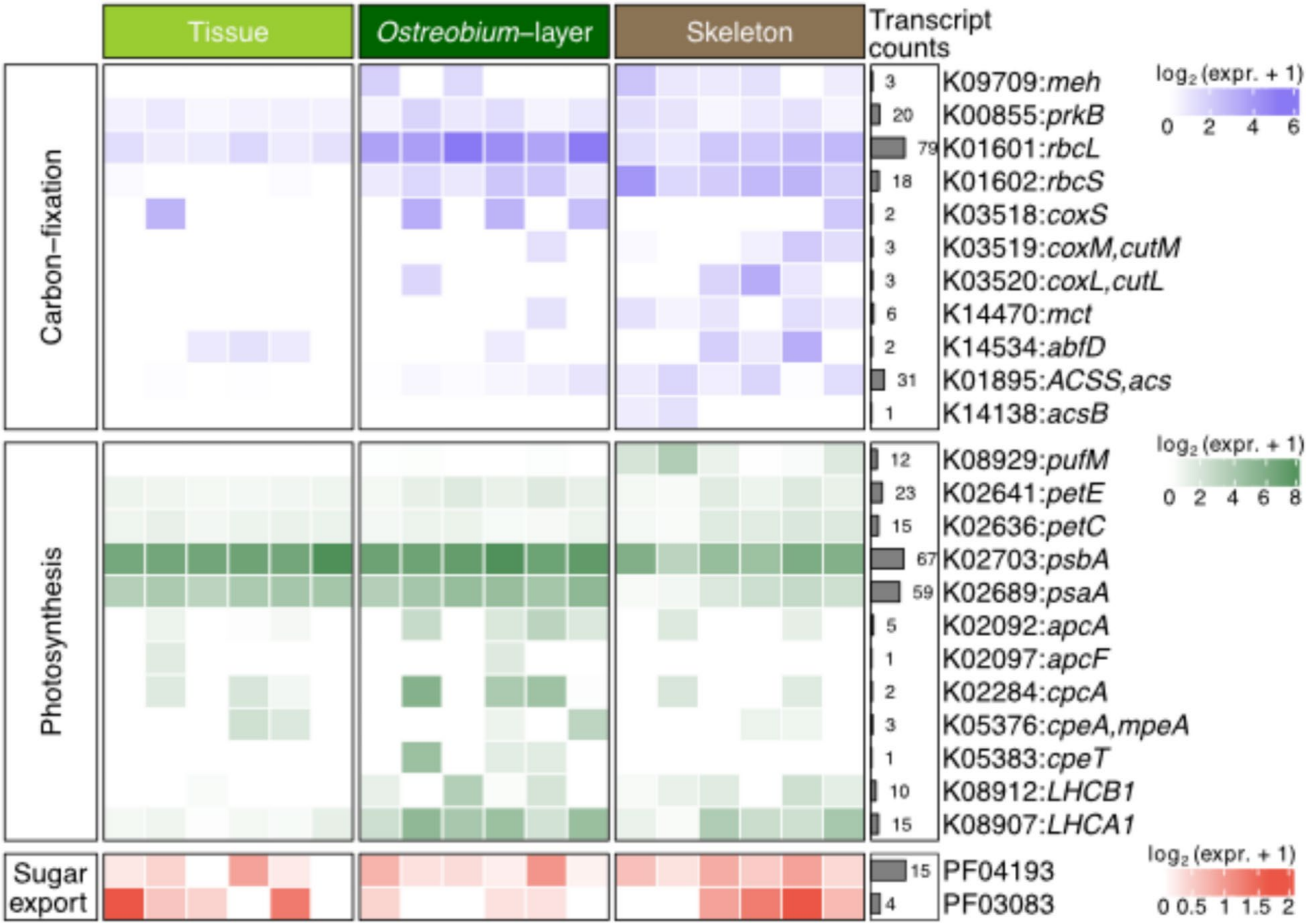
Transcriptomics profiles of carbon-fixation, photosynthesis and sugar export across coral compartments. Heatmap illustrating the expression levels (log_2_(expression + 1)) of marker genes across the Tissue, *Ostreobium *layer, and Skeleton. The horizontal bar plot (right) indicates the total transcript counts annotated for each identifier. Carbon-fixation: Genes include K09709: meh (3-methylfumaryl-CoA hydratase); K01601: rbcL and K01602: rbcS (ribulose-bisphosphate carboxylase large/small chains); K03518/19/20: coxS/M/L (carbon-monoxide dehydrogenase subunits); K14470: mct (malonyl-CoA:ACP transacylase); K14534: abfD (4-hydroxybutyryl-CoA dehydratase); K01895: ACSS/acs (acetyl-CoA synthetase); and K14138: acsB (acetyl-CoA synthase). Photosynthesis: Includes K08929: pufM (photosynthetic reaction center M subunit); K02641: petE(plastocyanin); K02636: petC (cytochrome—complex iron-sulfur subunit); K02703: psbA (photosystem II P680 reaction center D1 protein); K02689: psaA (photosystem I P700 chlorophyll apoprotein A1); and antenna proteins K02092: apcA (allophycocyanin alpha subunit), K02097: apcF (phycobilisome core component), K02284: cpcA(phycocyanin alpha chain), K05376: cpeA/mpeA (phycoerythrin alpha chain), K05383: cpeT (phycoerythrin-binding protein), K08912: LHCB1 (LHCII chlorophyll binding protein 1), and K08907: LHCA1 (LHCI chlorophyll binding protein 1). Sugar export: Represents Sugars Will Eventually Be Exported Transporters (SWEET) identified by conserved protein family (PF) domains PF04193 (PQ-loop repeat motif) and PF03083 (MtN3/saliva protein domain)

To characterize the functional landscape of the *P. lutea* holobiont, we specifically investigated pathways central to symbiotic maintenance and biogeochemical cycling, including photosynthesis, carbon fixation, sugar export, and the cycling of nitrogen and sulfur. These processes represent primary metabolic interactions between the host and its associated microbiome [77]. Our analysis identified the active expression of the Calvin-Benson-Bassham (CBB) cycle across all compartments, characterized by high transcript abundance for ribulose-1,5-bisphosphate carboxylase/oxygenase (RuBisCO) subunits (*rbcL:* 79 transcripts and *rbcS*: 18 transcripts) alongside Photosystem I (*psaA*: 59 transcripts) and Photosystem II (*psbA*: 67 transcripts) (Fig. 2). These genes were transcribed by a diverse consortium of phototrophs with distinct spatial distributions. *Symbiodiniaceae* dominated expression within the tissue, while Cyanobacteria were active across all compartments. Within the endolithic compartments (comprising both the *Ostreobium *layer and the deeper skeleton), the endolithic green alga *Ostreobium* and other *Ulvophyceae* were the primary contributors (Supplementary data file 2). While *rbcL* can be expressed by chemotrophs, our taxonomic annotations in this context identified exclusively photoautotrophic affiliations, suggesting that CBB-mediated fixation is driven by the photosynthetic community.

The observed expression patterns reflect specific microbial adaptations to the steep light gradients within the coral skeleton. Beyond core photosystems, we detected expression of *petC* (Rieske iron–sulfur protein) and *petE* (plastocyanin), supporting the presence of fully functional oxygenic photosynthetic electron transport chains. Notably, cyanobacteria-specific antenna proteins (*apcA, apcF, cpcA, cpeA, *and* cpeT*) were significantly more abundant in the *Ostreobium* layer and skeleton than in the tissue (Fig. 2). This spatial enrichment suggests that skeletal Cyanobacteria may utilize alternative chlorophylls (e.g., *d* and *f*) to capture far-red light that penetrates the coral tissue [78–80]. Simultaneously, *Ostreobium* expressed an expanded suite of light-harvesting complex (LHC) proteins and *Lhca1* gene duplicates, reflecting specialization for the low-light conditions of the endolithic niche [14, 81].

Within both the *Ostreobium *layer and the deeper skeleton, we identified metabolic versatility through alternative carbon fixation and energy-generating pathways. We detected expression of *coxL*, *coxM*, and *coxS,* affiliated with Cyanobacteria and Proteobacteria, suggesting the potential activity of carbon monoxide dehydrogenase (*CODH*), enabling CO oxidation and energy generation through chemosynthesis (Fig. 2). Additionally, transcripts for *acs* and *acsB,* encoding the acetyl-CoA synthase complex of the Wood–Ljungdahl pathway and anoxygenic photophosynthesis marker *pufM* were identified in the deeper skeleton, likely reflecting adaptations to anaerobic or low-oxygen microenvironments [13, 66].

Collectively, transcripts for photosynthetic antenna and CBB cycle accounted for more than 50% of the total carbon fixation pool with the endolithic compartments (comprising both the *Ostreobium *layer and the deeper skeleton). The robust co-expression of *rbcL/rbcS* with *psbA* and *psaA,* combined with the significantly higher expression levels relative to the Wood–Ljungdahl pathway (Fig. 2), identifies chlorophototrophy as the dominant carbon fixation strategy within the *P. lutea* holobiont. These results suggest that non-zooxanthellae phototrophs inhabiting the skeletal matrix constitute a substantial, yet understudied, potential source of organic carbon. While these transcriptomic profiles signal illustrate the functional potential for diverse carbon cycling, further experimental work is needed to quantify the actual metabolic fluxes and their specific contributions to coral productivity.

### Transcriptomic evidence for diverse eukaryotic sugar transporters beyond Symbiodiniaceae

To identify members of the coral holobiont potentially involved in the export of photosynthetically fixed carbon compounds (photosynthates), we focused on genes encoding SWEET (“Sugars Will Eventually be Exported Transporter”) proteins. SWEETs are bidirectional sugar uniporters that facilitate the diffusion of sugars such as glucose along concentration gradients across biological membranes [82, 83]. We identified transcripts encoding SWEET proteins, specifically associated with Pfam domains PF04193 and PF03083, across all three major compartments of the coral holobiont (Fig. 2). Taxonomic annotation revealed that the sugar transporters were predominantly eukaryotic, including Symbiodiniaceae, the endolithic alga *Ostreobium*, and unclassified green algae within the Chlorophyta (Supplementary data file 2). These organisms share a common genomic capacity for oxygenic photosynthesis via the Calvin cycle, in which glyceraldehyde-3-phosphate (G3P) serves as a precursor for sugar biosynthesis. Because SWEET-mediated transport is a passive process that is driven by a concentration gradient, the high rates of intracellular carbon fixation in these phototrophs could potentially create a source-to-sink gradient that favors the net export of glucose to the coral host and the broader heterotrophic microbial community [84]. This provides a putative mechanism for how eukaryotic phototrophs, both in the tissue and the skeletal compartment, may act as a primary carbon source for the heterotrophic members of the holobiont.

Interestingly, despite previous studies reporting sugar transporters in coral-associated bacteria [22], we did not detect transcripts encoding semiSWEET proteins, the prokaryotic homologs of SWEETs. This absence might reflect a biological partitioning of function, wherein eukaryotic symbionts serve as the primary carbohydrate exporters. Alternatively, it could be a result of detection limits or the divergence of bacterial sugar transporters from characterized semiSWEETs. Regardless, our findings highlight a potential central role for eukaryotes in sugar-based metabolic exchange among holobiont members.

Notably, we identified SWEET gene expression in *Ostreobium*, providing the first genomic and transcriptomic evidence consistent with sugar translocation by this skeleton-dwelling alga. While the bidirectional nature of SWEETs theoretically allows for flux in either direction, our finding of active sugar transporters provides a possible mechanistic explanation for previous radiolabeling studies [30, 31]. Earlier research using ^14^C and ^13^C-labeled photoassimilates demonstrated a net translocation of fixed carbon from endolithic algae into coral host lipids. By integrating these experimental observations with our detection of eukaryotic sugar uniporters, we propose a model where high intracellular glucose production in *Ostreobium* likely creates the concentration gradient necessary to drive the outward transport of photosynthates.

However, the bidirectional nature of these transporters also supports the potential for mixotrophy in *Ostreobium*. This suggests that endolithic algae might switch between exporting photosynthates and opportunistically scavenging exogenous sugars, potentially utilizing extracellular proteases to supplement carbon requirements depending on the steep light and nutrient gradients within the skeletal matrix. The ability of *Ostreobium* to maintain sugar transport machinery under thermal stress, even when Symbiodiniaceae populations are diminished, suggests a potential compensatory mechanism for carbon acquisition [30]. During bleaching events, this endolithic activity may provide an alternative source of photosynthate that may partially offset the loss of symbiont-derived carbon. While the net energetic benefit to the coral host remains to be quantified, the persistence of these pathways indicates that the skeletal matrix remains a metabolically active zone of potential carbon production during periods of holobiont stress.

In addition to phototrophs, we also detected the expression of SWEET-encoding transcripts in coral-associated fungi. This is the first direct molecular evidence suggesting fungal participation in sugar transport within the coral holobiont. However, unlike the phototrophic members, fungi are heterotrophic and do not fix carbon. Consequently, their participation in sugar transport likely reflects opportunistic scavenging or recycling of the host-derived sugars rather than the primary sugar export. Fungi are increasingly recognized for their roles in shaping coral-associated microbiomes and reef-scale biogeochemical cycling through nutrient processing [84], but without isotopic evidence of fungal-to-host carbon transfer, their involvement may reflect metabolic cross-feeding or carbon recycling rather than primary sugar export.

The identification of multiple eukaryotic lineages expressing genes associated with sugar translocation underscores the complex and potentially cooperative nature of carbon cycling within the coral holobiont. These sugar-transporting symbionts may play critical roles in sustaining coral energy homeostasis, although the directionality of flux likely depends on the metabolic status of the specific symbiont. Furthermore, this capability might also pose risks: Elevated extracellular sugar concentrations could unintentionally fuel the proliferation of opportunistic or pathogenic bacteria, potentially destabilize the microbial community and compromise coral health. Thus, while eukaryotic sugar transporters likely contribute to coral resilience, their activity may also influence disease susceptibility, highlighting the need for further investigation into the ecological and evolutionary consequences of carbon transfer dynamics in the coral holobiont.

### Potential for microbially mediated nitrogen metabolism within the skeleton

**Fig. 3:**
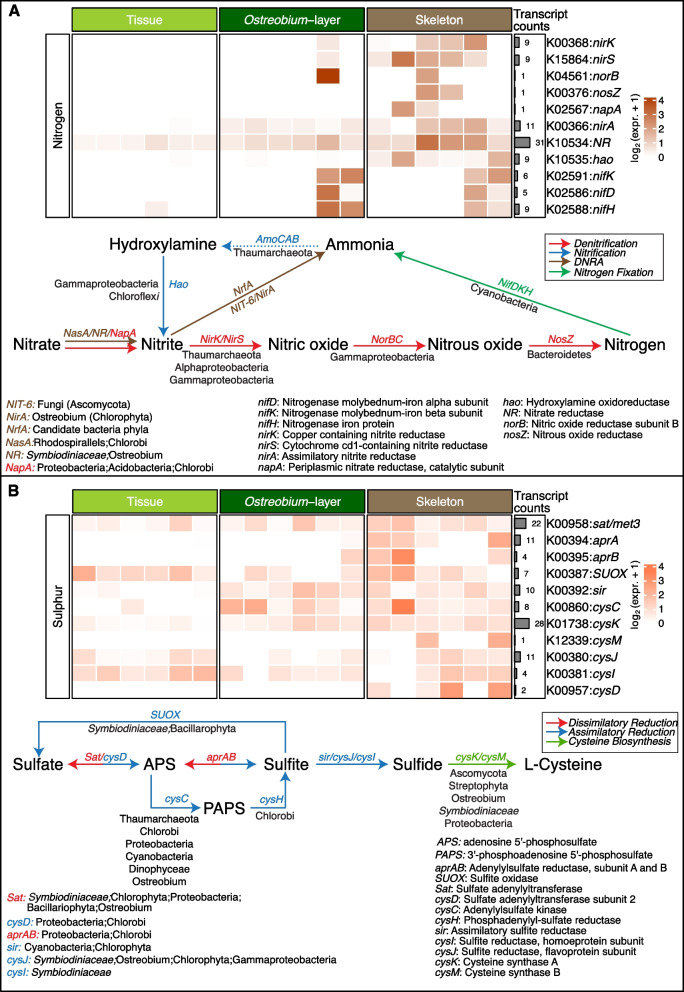
Transcriptome-based expression profiles of Nitrogen and Sulfur metabolism across coral compartments. **A** Heatmap showing the expression profiles of selected marker genes involved in nitrogen metabolism across coral compartments. The nitrogen cycle is illustrated, highlighting sub-pathways and contributing community members. **B** Heatmap showing the expression profiles of marker genes involved in sulfur metabolism across coral compartments, along with a bar plot indicating the count of transcripts. The sulfur metabolism pathway is illustrated, highlighting sub-pathways and contributing community members. The horizontal barplot shows the total number of transcripts annotated for that Kegg Ortholog across coral compartments. Expression values for both were averaged and log_2_(expression + 1) transformed

Our analysis revealed active expression of genes associated with nitrogen metabolism within the skeleton of *P. lutea*, suggesting a role for skeleton-associated microbes in sustaining nitrogen cycling within the coral holobiont. Expression of canonical nitrogen fixation marker genes *nifK, nifD*, and *nifH* was detected in both the *Ostreobium *layer and the deeper skeleton, pointing towards active nitrogen fixation in these predominantly anoxic microenvironments (Fig. 3A). Nitrogen cycling is essential in coral reef ecosystems, where nutrient scarcity demands tight microbial recycling to support coral growth and productivity [27]. While nitrogen-cycling microbes are widely documented in coral-associated microbiomes [27, 74], our study identified *Cyanobacteria* as the predominant taxonomically resolved nitrogen fixers in *P. lutea*. This contrasts with earlier reports emphasizing heterotrophic nitrogen-fixing bacteria [85–87] and instead aligns with recent genome-resolved metagenomic studies showing low diversity of nitrogen-fixing taxa in this species [20, 21]. It is important to note that a substantial proportion of transcripts remain unclassified, and additional nitrogen-fixing taxa may be present but undetected. Nonetheless, the predominance of cyanobacterial transcripts underscores their likely contribution to supporting nitrogen-limited growth of Symbiodiniaceae [8], and highlights their potential role in the coral holobiont’s nitrogen economy.

We also identified the transcriptomic signature of nitrification, the aerobic microbial process converting ammonia to hydroxylamine and subsequently to nitrite and nitrate, to be ubiquitous across all coral compartments (Fig. 3A). This process is vital for maintaining holobiont nutrient homeostasis and coral host physiology by facilitating ammonium regulation and internal biogeochemical nitrogen cycling. Specifically, nitrification may mitigate ammonia toxicity, as the excessive accumulation of metabolic ammonium can be deleterious to coral cellular function. Furthermore, it potentially facilitates nitrogen turnover, the rapid biological transformation of metabolic nitrogen waste (ammonium) into more oxidized inorganic forms (nitrite and nitrate). This turnover is thought to be critical in regulating internal nutrient availability; by converting ammonium, the holobiont may prevent the excessive nitrogen accumulation that could otherwise trigger an unbalanced proliferation of Symbiodiniaceae, a process known to destabilize the metabolic coupling between host and its symbionts [27]. Transcripts encoding nitrification-related genes were primarily assigned to Thaumarcheota, Gammaproteobacteria, and Chloroflexi, particularly in the deeper skeleton (Fig. 3A). These taxa likely contribute to ammonia oxidation, although unclassified transcripts and incomplete database coverage suggest that additional nitrifying microbes may be involved.

In contrast, denitrification was spatially restricted to the *Ostreobium *layer and deeper skeleton compartments, consistent with oxygen-limited conditions in these compartments [13, 88, 89]. We detected expression of key denitrification marker genes (*napA*, *nirS*, *norB*, and *nosZ*) in both eukaryotic phototrophs (Symbiodiniaceae, *Ostreobium*) and bacterial symbionts, including Rhodospirillales, Chlorobi, Proteobacteria, and Acidobacteria (Fig. 3A; Supplementary data file 3). The subsequent steps in denitrification, conversion of nitrite to nitric oxide, nitrous oxide, and nitrogen were largely attributed to Thaumarchaeota*,* and Proteobacteria (α- and γ-), and Bacteroidota. Importantly, we also detected fungal (Ascomycota) transcripts encoding *NIT-6* gene, involved in dissimilatory nitrate reduction to ammonium (DNRA), providing transcriptomic evidence for fungal contributions to nitrogen recycling within the holobiont.

The interplay between nitrification, denitrification, and DNRA, particularly within the skeleton, appears to be a likely mechanism crucial for maintaining nitrogen homeostasis. In the coral holobiont, this homeostasis is defined as the active regulation of internal nitrogen levels to keep Symbiodiniaceae in a state of nitrogen limitation, which ensures stable symbiont densities and efficient carbon translocation to the host [27]. DNRA may dominate under low-oxygen conditions (e.g., at night), recycling nitrate into ammonium when aerobic nitrification is less active [88, 89]. This could ensure stable ammonium availability, potentially supporting coral growth. In contrast, coupling of nitrification and denitrification may help regulate nitrogen levels under nutrient-rich conditions, preventing excessive proliferation of Symbiodiniaceae and maintaining a balanced holobiont metabolism.

Overall, our results expand current understanding of nitrogen cycling in coral holobionts by resolving the microbial taxa whose transcripts suggest active involvement in these processes. The coral skeleton emerges as a metabolically active zone for both aerobic and anaerobic nitrogen transformations, with stratified microbial communities potentially performing complementary functions. These findings underscore the likely importance of skeleton-associated microbes in holobiont nutrient dynamics and reef ecosystem functioning.

### Sulfur metabolizing microbes are widespread across coral compartments

Sulfur, abundant in seawater primarily as inorganic sulfate [90], is assimilated by the coral host, Symbiodiniaceae, and various members of the coral microbiome into essential organic compounds, making it a critical element for holobiont growth and function [91–93]. In this study, we identified expression of transcripts encoding sulfur cycling marker genes across all three coral compartments (Fig. 3B), underscoring the distributed nature of sulfur metabolism within the holobiont. Notably, transcript abundance for most sulfur-related genes was highest in the skeleton, suggesting a significant role for the endolithic microbiome in coral sulfur cycling.

We detected expression of anaerobic dissimilatory sulfate reduction (DSR) marker genes, adenosine-5’–phosphosulfate reductase alpha subunit (*aprA*) and beta subunit (*aprB*), exclusively in the skeleton compartment. Taxonomic annotation identified these transcripts as originating from canonical sulfate-reducing bacteria (SRB), including Deltaproteobacteria, as well as anoxygenic phototrophic Chlorobi (Fig. 3B). Although Chlorobi carry the *aprAB* genes, they typically participate in sulfur oxidation rather than reduction [94, 95], and their presence here suggests diverse sulfur redox processes within the skeleton. This observation supports prior metabarcoding and metagenomic studies, which reported abundant SRBs and assembled their genomes from the skeleton of *P. lutea* and *Isopora palifera* [20]. The co-detection of SRBs and Chlorobi within the same compartment aligns with previous reports of steep vertical oxygen gradients and formation of anoxic microniches in coral skeletons [13, 20, 23, 88]. Limited gas diffusion imposed by dense coral tissue and the overlying mucus layer, combined with high rates of respiratory oxygen consumption by the holobiont, particularly at night, can lead to rapid oxygen depletion within the skeletal matrix [13, 80, 89]. Furthermore, microsensor measurements of light penetration into the coral skeleton have shown that near-infrared light, the wavelength utilized by anoxygenic phototrophs, penetrates efficiently into the deeper skeletal layers [80]. This physical availability of light is corroborated by hyperspectral imaging, which provides direct evidence of the presence of bacteriochlorophylls within the skeletal microbiome [13]. Under these conditions, the simultaneous detection of SRB and anoxygenic phototrophic Chlorobi suggests a syntrophic sulfur-based interaction. In this model, sulfide generated by sulfate reduction serves as an electron donor for phototrophic metabolism, effectively coupling anaerobic mineralization with light-driven carbon fixation across microscale redox gradients. Such metabolic coupling has been observed in microbial mats, stratified lake waters, and coral skeletons [23, 96–98]. The simultaneous expression of *aprAB* genes by both groups in this study provides molecular evidence for this syntropy within the coral holobiont.

We also identified a complex and spatially distributed expression pattern of assimilatory sulfate reduction (ASR) genes. Transcripts for adenylylsulfate kinase (*cysC*), the sulfite reductase subunits (*cysI* and *cysJ*), and sulfite oxidase (*SUOX)* were detected across all compartments, whereas sulfate adenylyltransferase (*cysD*) expression was restricted to the deeper skeleton (Fig. 3B). The gene encoding cysteine kinase (*cysK*), which links ASR to L-cysteine biosynthesis, was consistently expressed in all compartments. These findings suggest widespread ASR activity throughout the holobiont, potentially supporting sulfur assimilation and amino acid biosynthesis. Taxonomic analysis of ASR gene transcripts revealed a diverse array of eukaryotic and prokaryotic symbionts. These included microeukaryotes such as Symbiodiniaceae, *Ostreobium*, and other Chlorophyta, as well as bacteria including Cyanobacteria, Thaumarchaeota, and Proteobacteria (Fig. 3B). Notably, all ASR transcripts were exclusively assigned to algae and bacteria, consistent with the known distribution of this pathway among plants, algae, and microbes [99].

Together, these results provide new insights into the compartmentalized and potentially microbially mediated sulfur cycling processes within *P. lutea*. The coral skeleton emerges as a distinct biogeochemical hotspot, where both dissimilatory and assimilatory sulfur transformations likely occur in proximity, potentially driven by specialized microbial consortia. The transcriptomics evidence of potential syntrophic sulfur interactions between SRBs and Chlorobi highlights the complexity of this anaerobic niche, where the metabolic waste of one group might sustain the requirements of another. The coral skeleton thus functions as a dynamic biogeochemical compartment characterized by an active sulfur cycle potential, the full implications of which remain to be elucidated.

### A diverse repertoire of micro-eukaryotic ROS and RNS scavenging homologs

**Fig. 4:**
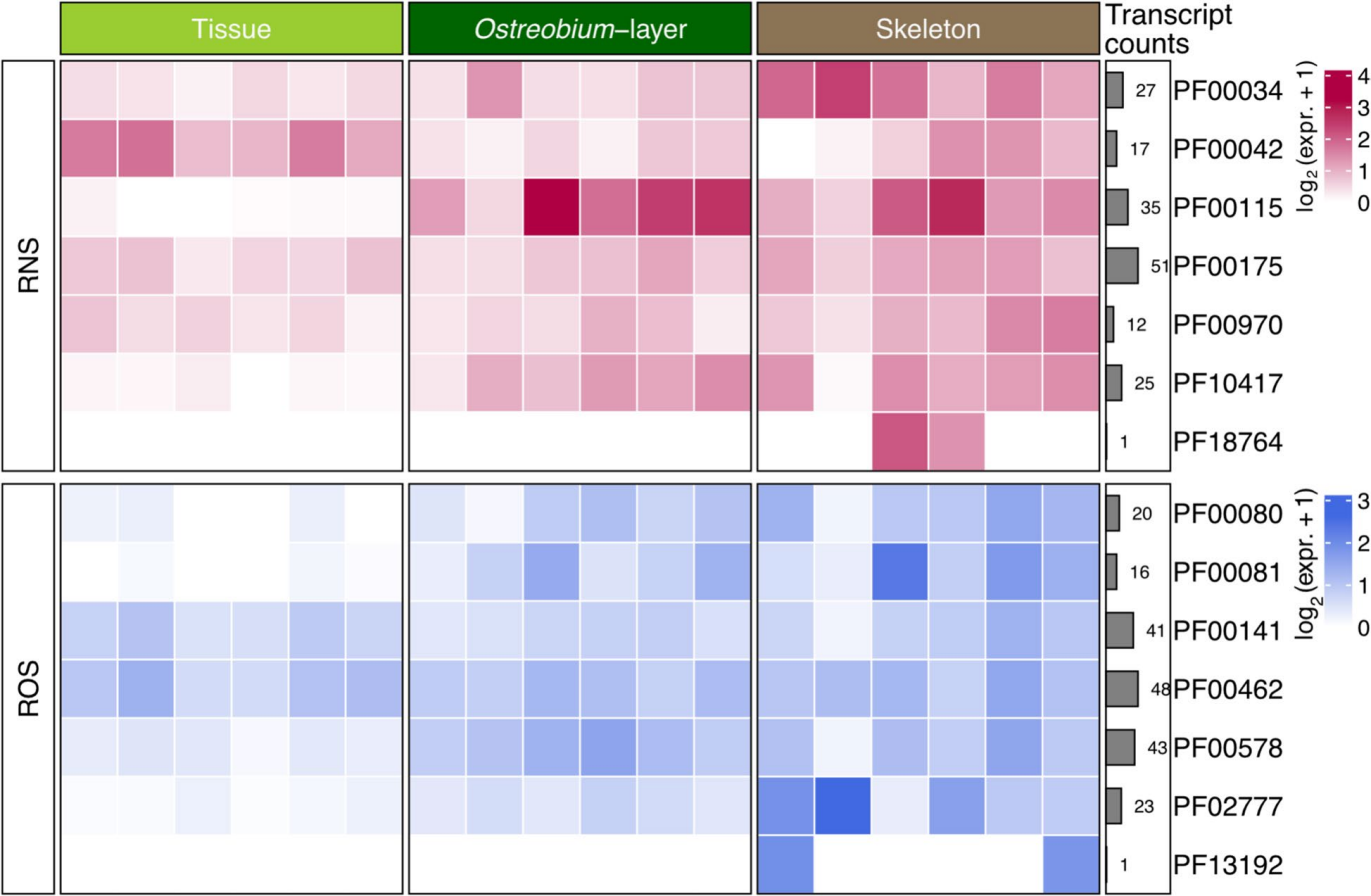
Transcriptome-based expression profiles of reactive oxygen species (ROS) and reactive nitrogen species (RNS) marker genes across coral compartments. Heatmap displaying the expression of different protein families. The histogram shows the count of transcripts for each protein family involved in ROS and RNS scavenging across coral compartments. PF00034:Cytochrome C domain, PF00042: Globin, PF00115: Cytochrome C and Quinol oxidase polypeptide I, PF00175: Oxidoreductase NAD binding domain, PF00970: Oxidoreductase FAB binding domain, PF10417: C-terminal domain of 1-Cys peroxiredoxin, PF18764: Nitrous oxide reductase propeller repeats. Expression values for both were averaged and log_2_(expression + 1) transformed

Coral reefs are facing unprecedented threats due to rising sea surface temperatures, resulting in more frequent and prolonged coral bleaching events [100]. Most theories on the onset of coral bleaching suggest that the overproduction of reactive oxygen species (ROS) and reactive nitrogen species (RNS) is a key trigger, leading to cellular damage and stress under adverse environmental conditions [101]. However, mounting evidence suggests that bleaching onset is a multi-factorial process; for instance, disruptions in nitrogen cycling and the loss of nitrogen limitation within the symbiont niche may play a critical role in the breakdown of the holobiont relationship [102]. Consequently, rapid scavenging of ROS and RNS, coupled with the maintenance of nutrient exchange, is crucial in maintaining homeostasis and protecting the coral holobiont in stressed and healthy corals. We observed widespread expression of seven protein families with functions related to ROS or RNS scavenging across the tissue, *Ostreobium *layer, and deeper skeleton compartments. Specifically, transcripts encoding for genes involved in RNS scavenging, including nitric oxide reductases (PF00175: *hmp*; PF00115, and PF00034: *norBC*) and peroxynitrite to nitrate reduction (PF10417: *ahpC*), were expressed in all compartments (Fig. 4). A single transcript encoding nitrous oxide reductase (PF18764: *nosZ*) was identified and expressed exclusively in deeper skeleton samples. This enzyme catalyzes the last step of the dissimilatory metabolic pathway of denitrifying bacteria, which were abundant in the deeper skeleton. Similar observations were made for ROS scavenging expression profiles. We identified 48, 43, and 41 transcripts encoding the general ROS scavenger Glutaredoxin (PF00462), as well as hydrogen peroxide scavengers Peroxiredoxins (PF00578) and peroxidases (PF00141). These transcripts were expressed in all coral compartments along with transcripts encoding superoxide dismutases (PF00081 and PF02777: SOD Mn and PF00080: SOD cu–zn) (Fig. 4). The diversity of ROS and RNS scavenging transcripts was dominated by sequences of micro-eukaryotic origin (76% of unique homologs)**,** including Symbiodiniaceae, *Ostreobium*, Chlorophyta, and Fungi. The remaining 24% of unique scavenging homologs attributed to archaeal and bacterial sources, encompassing Thaumarchaeota, Proteobacteria (Rhodospirillales, Xanthomonadales), Cyanobacteria, Chlorobi, Chloroflexi, and Bacteroidetes (*Candidatus* Amoebophilus) (Supplementary data file 4).

Scavenging ROS and RNS is a key selection criterion for coral probiotics, as emphasized by recent studies identifying potential beneficial bacteria for corals [2, 22, 103–105]. However, our finding that the highest diversity of unique ROS and RNS scavenging homologs originates from micro-eukaryotic inhabitants of the coral holobiont rather than in bacteria raises critical questions regarding which organisms serve as the primary drivers of antioxidant defense under thermal stress. While bacteria are essential, the expansive micro-eukaryotic scavenging repertoire suggests the current probiotic strategies, which are primarily focused on bacterial taxa, may overlook the most versatile components of the holobiont’s stress-response system. These findings underscore the need for functional studies to validate the contribution of specific micro-eukaryotic isoforms, which could guide the development of more comprehensive, multi-kingdom consortia to enhance coral thermal tolerance.

### Methodological considerations in holobiont meta-transcriptomics

The simultaneous sequencing of rRNA and mRNA used in this study provides a synchronized link between community structure and functional activity within a biological sample. However, using total RNA-seq without an explicit rRNA depletion step introduces interpretative trade-offs. Ribosomal RNA typically constitutes > 90% of total RNA, thereby reducing the effective sequencing depth available for the protein-coding mRNA pool relative to rRNA-depleted libraries [106]. In our dataset, this was particularly evident in the tissue compartment, where host-related rRNA dominated. As noted in the Supplementary Table S1, the total number of recovered mRNA reads is relatively small, which represents a key limitation of this baseline study. This depth constraint likely limited the detection of low-abundance transcripts associated with transient signaling or specialized metabolic activity [107].

Despite these limitations, avoiding rRNA depletion prevented the off-target loss of mRNA or the preferential depletion of specific microbial taxa [106]. Furthermore, rRNA and mRNA differ substantially in molecular stability, with rRNA generally exhibiting longer half-lives than the more rapidly turned-over mRNA pool [108, 109]. By retaining both RNA fractions, our approach enabled a direct, sample-matched comparison between taxonomic potential and contemporaneous transcriptional activity, thereby minimizing the ’linkage bias’ often encountered when comparing decoupled metagenomic and meta-transcriptomic datasets [110].

To overcome the reduced mRNA yield inherent to total RNA sequencing, we sequenced deeply across compartments (~ 23 million reads). While this sequencing effort was essential for supporting the compartment-resolved interpretations presented in this manuscript, the resultant data should be viewed as a foundational functional profile. Our findings, including evidence for substantial functional redundancy across carbon, nitrogen, and sulfur cycling pathways, transcriptional signatures consistent with fixed-carbon transfer from *Ostreobium* to other holobiont members (including the host), and stress-mitigation functions such as scavenging of reactive oxygen and nitrogen species distributed across diverse holobiont members, provide a robust baseline for the most active metabolic processes. However, deeper sequencing or targeted enrichment will be necessary in future studies to fully elucidate the rare or low-expression components of the holobiont’s functional landscape.

## Conclusion

**Fig. 5:**
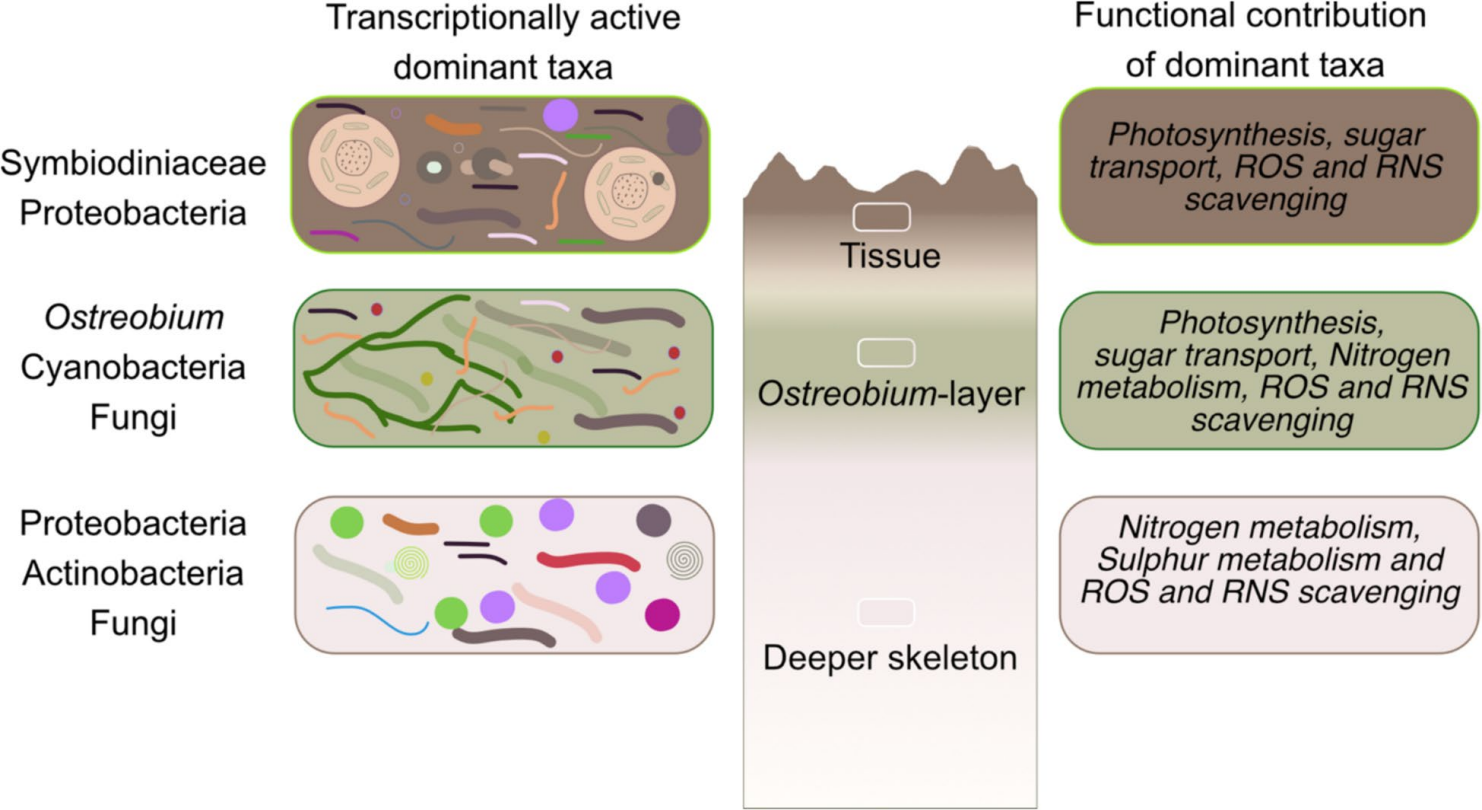
Overview of the transcriptionally dominant taxa and their functional contribution in different compartments of the coral identified in this study

This study provides novel insights into the transcriptionally active communities within the coral holobiont, mapping their functional potential across distinct ecological compartments: coral tissue, the endolithic *Ostreobium *layer, and the deeper skeleton layer. Our analysis revealed a nuanced stratification of microbial activity, where microeukaryotes dominate key metabolic processes in the coral tissue and *Ostreobium *layer, while prokaryotes emerge as the primary active symbionts in the deeper skeleton (Fig. 5). These findings underscore how physicochemical gradients, including light penetration, nutrient availability, and carbon distribution, sculpt both community composition and functional specialization.

Functional analysis highlighted the critical contributions of Symbiodiniaceae,* Ostreobium*, and Cyanobacteria to photosynthate production with Symbiodiniaceae predominantly occupying the coral tissue, while *Ostreobium* and Cyanobacteria play pivotal roles in the endolithic *Ostreobium *layer (Fig. 5). These symbionts, evolutionarily adapted to varying light conditions and utilizing distinct photosynthetic pigments, demonstrated unique nutrient cycling capabilities. Notably, in addition to Symbiodiniaceae, we identified *Ostreobium* and fungi as the only taxa capable of transporting sugars via SWEET proteins, providing a new molecular basis for understanding the potential nutrient exchange mechanisms within the holobiont. The identification of Cyanobacteria as the exclusive nitrogen fixers, alongside a diverse repertoire of micro-eukaryotic ROS and RNS scavenging homologs, suggests a complex division of labor that likely contributes to maintaining holobiont homeostasis. While this research advances our understanding of these internal processes, it highlights the necessity for high-resolution niche mapping. Future investigations should employ microscale sampling, such as laser capture microdissection or oxygen-sensitive optodes, combined with single-cell transcriptomics to more precisely map metabolic signaling between hosts and symbionts [10, 111, 112]. Ultimately, these insights provide a foundational framework for understanding the coral skeleton as a metabolically active environment. By characterizing the biogeochemical contributions and genomic toolkits of understudied endolithic holobiont members, this study shifts the focus toward a more inclusive, multi-kingdom view of coral microbial ecology, essential for predicting how the holobiont functions under environmental change.

## Supplementary Information

Supplementary Material 1.

Supplementary Material 2.

Supplementary Material 3.

Supplementary Material 4.

Supplementary Material 5.

Supplementary Material 6.

Supplementary Material 7.

Supplementary Material 8.

Supplementary Material 9.

## Data Availability

Raw reads generated in this study have been submitted to NCBI SRA under the Bioproject PRJNA1207708. All codes and files required to regenerate the figures have been made available here https://github.com/kshitijtandon/Plutea_metatranscriptome.
