# Supplementary material for: RNA-seq sheds light on “who is doing what” in the coral Porites lutea
Source: Microbiome. 2026 May 2;14:169. doi: 10.1186/s40168-026-02414-9 (PMC13289395; doi:10.1186/s40168-026-02414-9)
Supplement: Supplementary file 2 — Supplementary Material 1. [file 40168_2026_2414_MOESM1_ESM.docx]

**Using TRIzol (Invitrogen) with BCP and glycogen**

**Preparation**:

mortar and pestle, 1.5 ml tubes, 2 ml tubes

liquid nitrogen

BCP, isopropanol, high-salt solution, RNase-free water, 75% ethanol, 2M sodium chloride.

**High-salt solution (**NaCl 1.2 M + Sodium citrate 0.8 M**)**

For 30ml HSS: NaCl 2.1038g, Na_3_C_6_H_5_O_7_·2H_2_O 7.0584g

**Lyse samples and separate phases**

1. Add 1 ml of TRIzol reagent per 50-100 mg of tissue to the sample and vortex, put on ice for 5 to 10 min.
2. Centrifuge the lysate for 10 minutes at 12,000 × g at 4°C, then transfer the clear supernatant (almost 1 mL) to a new tube.
3. Incubate on ice for 5 minutes to allow complete dissociation of the nucleoproteins complex.
4. Add 0.1 mL of BCP per 1 mL of TRIzol™ Reagent used for lysis, securely cap the tube, then vigorously mix by shaking.
5. Incubate for 15 minutes on ice.
6. Centrifuge the sample for 15 minutes at 12,000 × g at 4°C.

Note: The mixture separates into a lower red phenol-chloroform, an interphase, and a colorless upper aqueous phase.

1. Transfer the aqueous phase (500-600 μl) containing the RNA to a new tube by angling the tube at 45° and pipetting the solution out.

**Isolate RNA**

**Precipitate the RNA**

(Optional step) Add 1/10 volume of 2M sodium chloride (from glycogen protocol).

1. Add 1 µL (Thermo Scientific) of RNase-free glycogen as a carrier to the aqueous phase.
2. Add 0.25 mL of isopropanol and 0.25 mL high salt solution (if there is no HHS, add 0.5 mL isopropanol instead) to the aqueous phase and mix by inverting, per 1 mL of TRIzol™ Reagent used for lysis.
3. Incubate for 30 minutes at -80°C (longer incubation time and lower temperature provide better recovery of nucleic acids).
4. Centrifuge for 10 minutes at 12,000 × g at 4°C.

Note: Total RNA precipitate is invisible and forms a white gel-like pellet at the bottom of the tube.

1. Discard the supernatant with a micropipette.

**Prepare 75% ethanol.**

**Wash the RNA**

1. Resuspend the pellet in 1 mL of **cold** 75% ethanol per 1 mL of TRIzol™ Reagent used for lysis.

Note: The RNA can be stored in 75% ethanol for at least 1 year at –20°C, or at least 1 week at 4°C.

1. Vortex the sample brieﬂy then centrifuge for 5 minutes at 7500 × g at 4°C.
2. Discard the supernatant with a micropipette.
3. Vacuum or air dry the RNA pellet for 5–10 minutes. Don’t do this with vacuum centrifuge.

**Solubilize the RNA**

Resuspend the pellet in 20–50 µL of RNase-free water by pipetting up and down. Add 1 µL RNase inhibitor for long-term storage.

**Supplementary Figures legends**

**Supplementary Fig. S1.** Principle Coordinate Analysis plots, for rRNA and mRNA level at sub-replicate level. The three compartments (Tissue, Ostreobium-layer, and Skeleton) Replicates (A or B) from the same colony and layer cluster closely together, indicating high technical reproducibility. No distinct clustering was observed based on the colonies collected in day (colonies 1, 3, 4 and 5) and vs nighttime (Colonies 6 and 7).

**Supplementary Fig. S2.** Barplots showing rRNA based relative abundance profile of prokaryotic and eukaryotic species in *Porites lutea* **A)** Tissue, **B)** Ostreobium-layer **C)** Skeleton layer.

**Supplementary Fig. S3.** Barplots showing meta-transcriptome based relative abundance profile of prokaryotic and eukaryotic species in *Porites lutea* **A)** Tissue, **B)** Ostreobium-layer **C)** Skeleton layer.

**Supplementary Fig. S4.** Plot to show the count of Cluster of orthologs based functional categories annotated across *Porites lutea* tissue, Ostreobium and skeleton layer.

**Supplementary Table S1.** Summary of reads at sub-replicates level.
