# Supplementary figures and images for: RNA-seq sheds light on “who is doing what” in the coral Porites lutea
Source: Microbiome. 2026 May 2;14:169. doi: 10.1186/s40168-026-02414-9 (PMC13289395; doi:10.1186/s40168-026-02414-9)

rRNA level

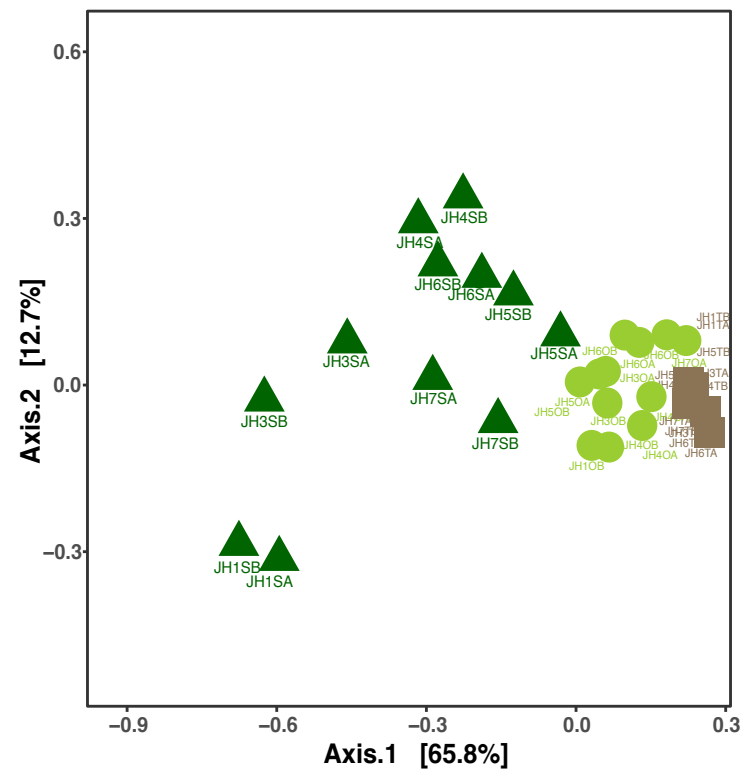

mRNA level

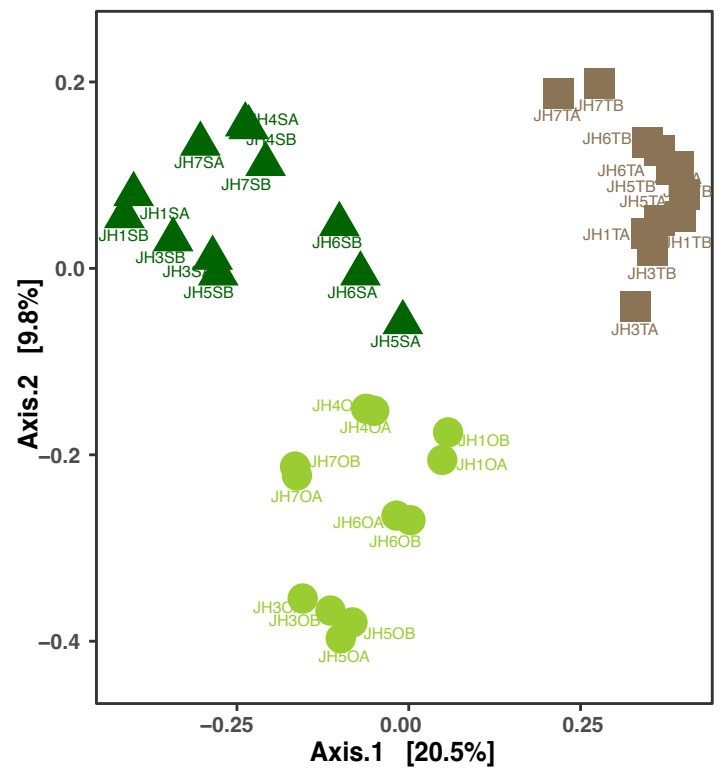

Compartment

Tissue    Osteobium-layer    Skeleton

Supplement: Supplementary file 6 — Supplementary Material 5. [file 40168_2026_2414_MOESM5_ESM.pdf]

rRNA based abundance profiles

A

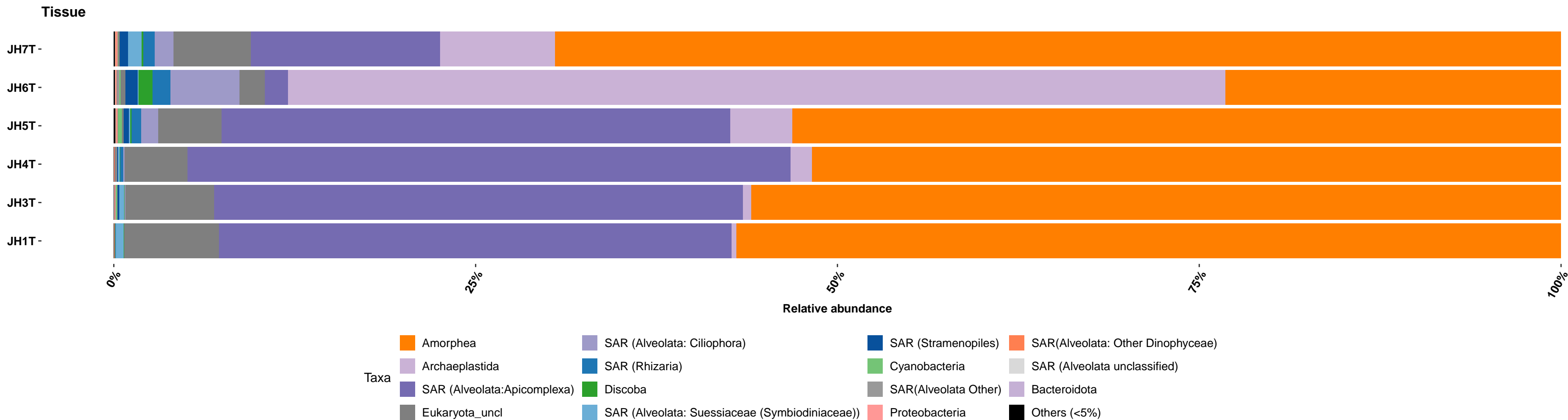

B

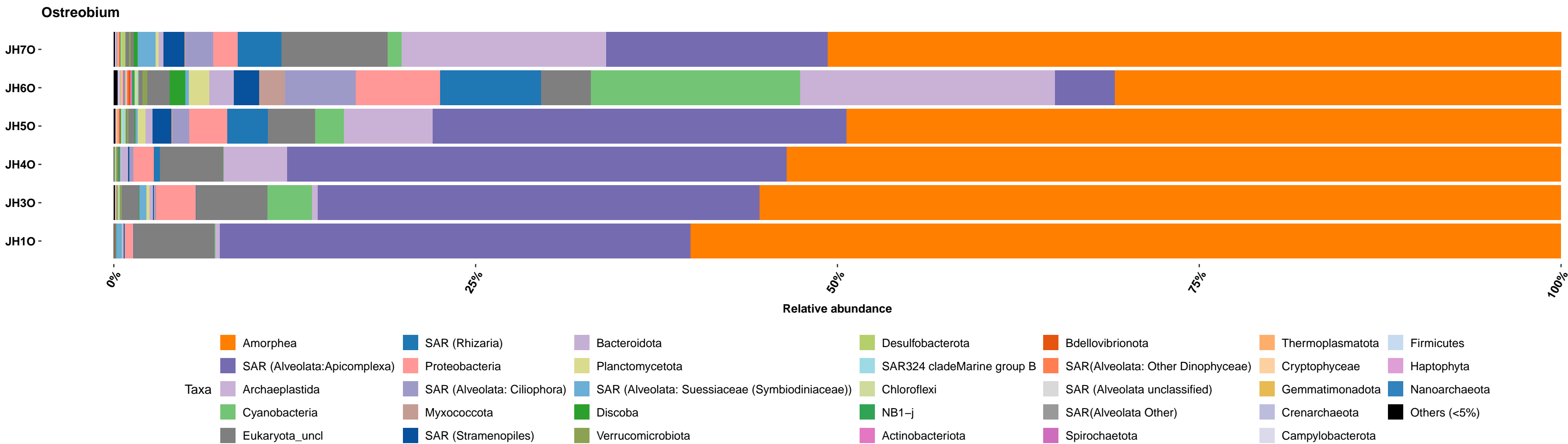

C

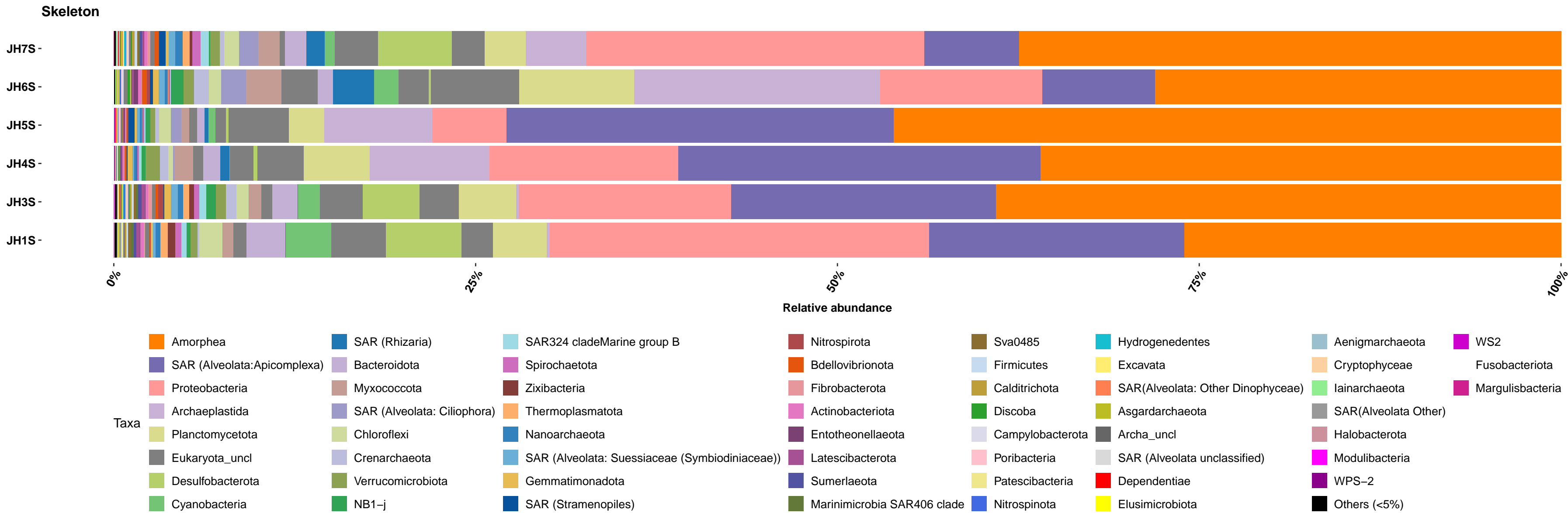

Supplement: Supplementary file 7 — Supplementary Material 6. [file 40168_2026_2414_MOESM6_ESM.pdf]

Meta-transcriptome based abundance profiles

A

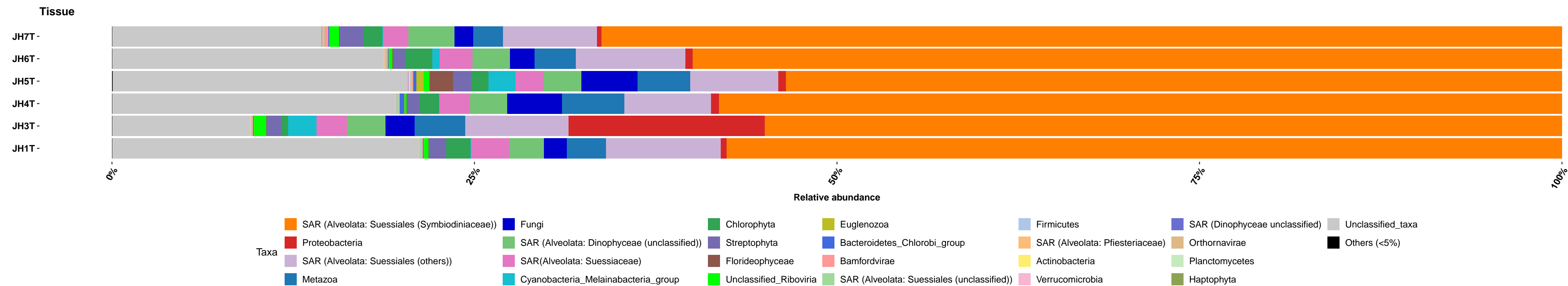

B

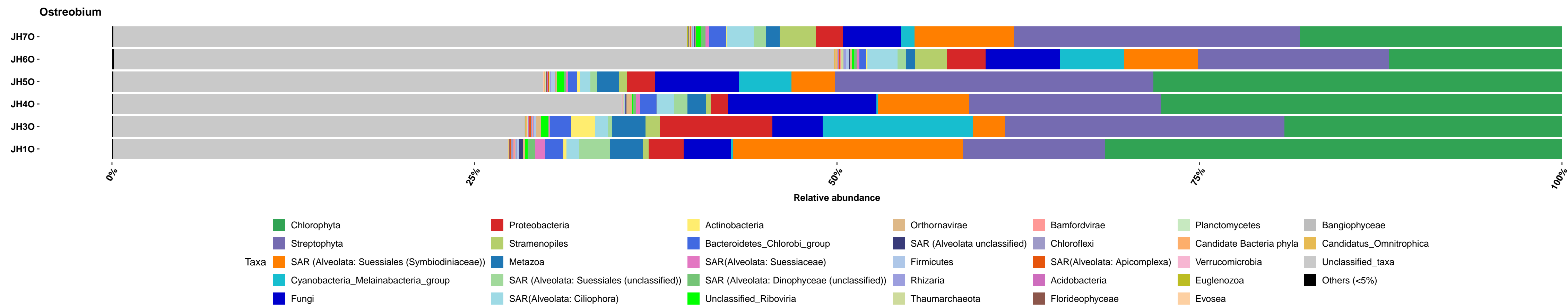

C

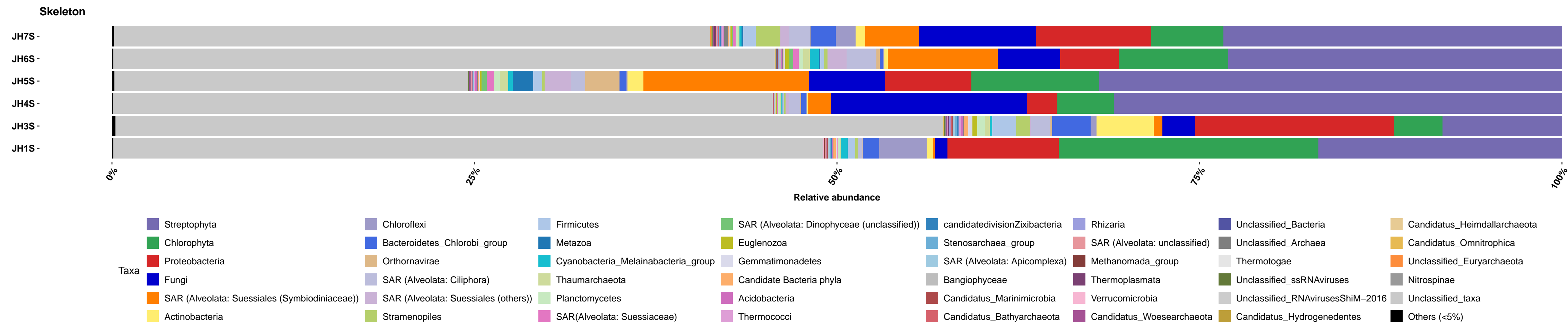

Supplement: Supplementary file 8 — Supplementary Material 7. [file 40168_2026_2414_MOESM7_ESM.pdf]

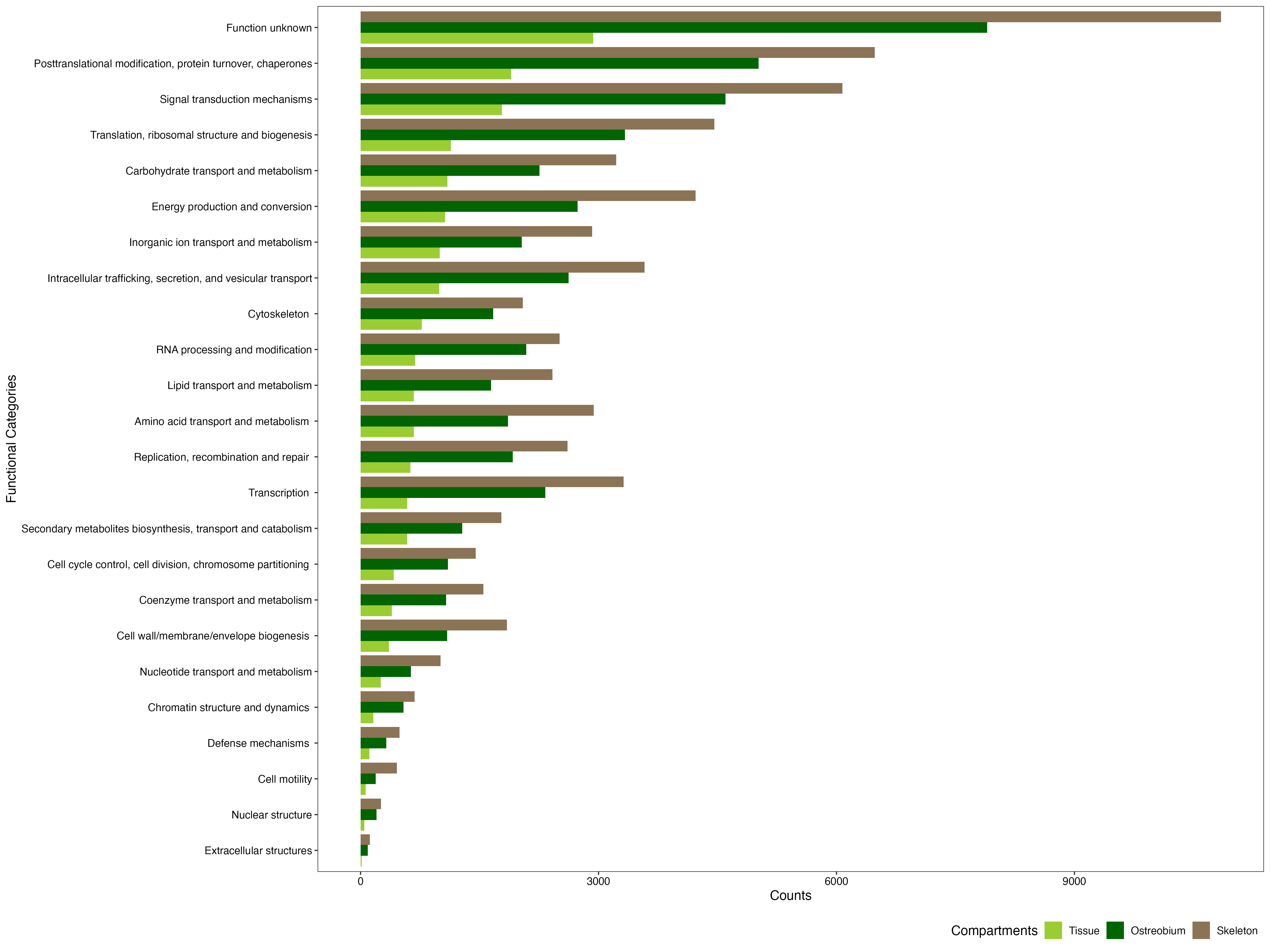

Supplement: Supplementary file 9 — Supplementary Material 8. [file 40168_2026_2414_MOESM8_ESM.png]
